# *7-UP:* Generating in silico CODEX from a small set of immunofluorescence markers

**DOI:** 10.1093/pnasnexus/pgad171

**Published:** 2023-05-19

**Authors:** Eric Wu, Alexandro E Trevino, Zhenqin Wu, Kyle Swanson, Honesty J Kim, H Blaize D’Angio, Ryan Preska, Aaron E Chiou, Gregory W Charville, Piero Dalerba, Umamaheswar Duvvuri, Alexander D Colevas, Jelena Levi, Nikita Bedi, Serena Chang, John Sunwoo, Ann Marie Egloff, Ravindra Uppaluri, Aaron T Mayer, James Zou

**Affiliations:** Enable Medicine, Menlo Park, CA 94025, USA; Department of Electrical Engineering, Stanford University, Stanford, CA 94305, USA; Enable Medicine, Menlo Park, CA 94025, USA; Enable Medicine, Menlo Park, CA 94025, USA; Department of Chemistry, Stanford University, Stanford, CA 94305, USA; Department of Computer Science, Stanford University, Stanford, CA 94305, USA; Enable Medicine, Menlo Park, CA 94025, USA; Enable Medicine, Menlo Park, CA 94025, USA; Enable Medicine, Menlo Park, CA 94025, USA; Enable Medicine, Menlo Park, CA 94025, USA; Department of Pathology, Stanford University, Stanford, CA 94305, USA; Department of Pathology and Cell Biology, Columbia University, New York, NY 10027, USA; Department of Otolaryngology, University of Pittsburgh, Pittsburgh, PA 15213, USA; CellSight Technologies, San Francisco, CA 94107, USA; CellSight Technologies, San Francisco, CA 94107, USA; Department of Otolaryngology-Head and Neck Surgery, Stanford University, Stanford, CA 94305, USA; Department of Otolaryngology-Head and Neck Surgery, Stanford University, Stanford, CA 94305, USA; Department of Otolaryngology-Head and Neck Surgery, Stanford University, Stanford, CA 94305, USA; Department of Medical Oncology, Dana-Farber Cancer Institute, Boston, MA 02215, USA; Department of Medical Oncology, Dana-Farber Cancer Institute, Boston, MA 02215, USA; Enable Medicine, Menlo Park, CA 94025, USA; Enable Medicine, Menlo Park, CA 94025, USA; Department of Electrical Engineering, Stanford University, Stanford, CA 94305, USA; Department of Computer Science, Stanford University, Stanford, CA 94305, USA; Department of Biomedical Data Science, Stanford University, Stanford, CA 94305, USA

## Abstract

Multiplex immunofluorescence (mIF) assays multiple protein biomarkers on a single tissue section. Recently, high-plex CODEX (co-detection by indexing) systems enable simultaneous imaging of 40+ protein biomarkers, unlocking more detailed molecular phenotyping, leading to richer insights into cellular interactions and disease. However, high-plex data can be slower and more costly to collect, limiting its applications, especially in clinical settings. We propose a machine learning framework, *7-UP*, that can computationally generate in silico 40-plex CODEX at single-cell resolution from a standard 7-plex mIF panel by leveraging cellular morphology. We demonstrate the usefulness of the imputed biomarkers in accurately classifying cell types and predicting patient survival outcomes. Furthermore, *7-UP*'s imputations generalize well across samples from different clinical sites and cancer types. *7-UP* opens the possibility of in silico CODEX, making insights from high-plex mIF more widely available.

**Significance Statement**

Multiplex immunofluorescence imaging is a powerful approach for studying spatial proteomics. However such experimental data are still challenging and expensive to generate. In this paper, we propose 7-UP, a machine learning algorithm to computationally generate high-resolution immunofluorescence images of up to 40 different antibodies on the same tissue section. 7-UP uses cellular morphology and a small number of antibody stains to impute the abundance of the other antibody stains. We demonstrate its applications across diverse human tumor samples. 7-UP's imputations generalize well across samples from different clinical sites and cancer types.

## Introduction

The tissue microenvironment (TME) is a complex milieu comprising many cell types and heterogeneous cell states. Common techniques for understanding the TME like mass spectrometry (1) and flow cytometry (2) allow for bulk measurements of many cell biomarkers but discard valuable spatial information in the process. Recently, multiplexed molecular imaging assays have enabled the quantification of cell types and molecules in their native tissue context. Commercial multiplex immunofluorescence (mIF) systems are increasingly commonplace in clinical diagnostic and prognostic settings (3) but are typically limited to quantifying between one and seven biomarkers (4).

More recently, mIF techniques such as co-detection by indexing (CODEX) (5) quantify 40 or more markers in situ, allowing a richer and more holistic characterization of the TME and its underlying cell types and disease processes. However, CODEX systems are significantly more costly and time-consuming to run when compared to most low-plex systems, which limits their wider adoption in clinical settings.

To address this limitation, we introduce *7-UP*, a machine learning framework that generates in silico high-plex mIF (30+ biomarkers) from only a panel of seven experimentally measured biomarkers. Whereas typical 7-plex measurements can only resolve up to five to seven distinct cell types (3), using the imputed markers from *7-UP* enables the identification of up to 16 cell types. Moreover, the imputed biomarker expressions can predict complex clinical outcomes with accuracy comparable to using experimental measurements from CODEX. *7-UP* generalizes to new cancer types and samples that come from different clinical sites than its training data. Our approach highlights a significant opportunity to use machine learning toward inferring high-dimensional molecular features from commonly available low-plex imaging data.

Imputation techniques have been applied to missing data in genomics (6–8) and transcriptomics (9, 10) data sets, as well as in mass spectrometry and shotgun proteomics (6, 11, 12) data. Deep learning has been used to extract morphological and spatial features from pathology H&E-stained slides (13–16) and, in turn, enabled in silico IHC staining (17) and spatial transcriptomics (18). More recently, computational methods have been developed for improving cell-type classification in CODEX-acquired data (19) and augmenting with spatial information in particular (20). To date, our work is the first to demonstrate the effectiveness of deep learning–based morphological feature extraction toward mIF imputation.

## Results

### 7-UP summary

**Fig. 1.**
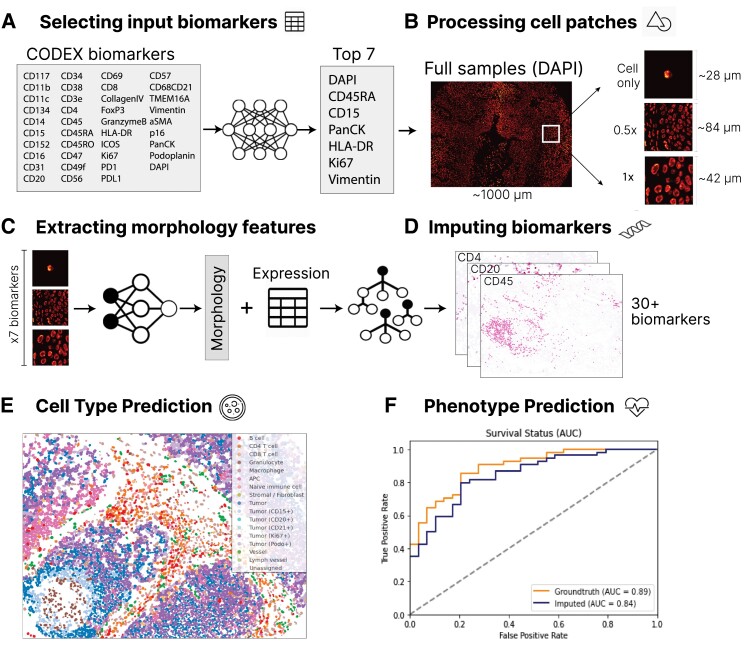
Overview of the *7-UP* Framework. (**Panel A**) From the full CODEX panel of biomarkers, a concrete autoencoder selects the seven biomarkers that are best able to reconstruct the full panel. (**Panel B**) From a full sample (∼1,000 microns wide), image patches are extracted for each cell: *cell only*, containing the morphology of the cell at 3× scaling, *0.5*×, a ∼84-micron neighborhood around the cell, and 1×, a ∼42-micron neighborhood around the cell. (**Panels C and D**) Each cell has 3 patches produced for each of the 7 biomarkers, totaling 21 patches used as input to a deep learning model. This model extracts morphological features for each cell, which are combined with the average expressions of the top seven biomarkers to predict the average expressions of the remaining CODEX panel biomarkers using a machine learning regression model. (**Panel E**) The imputed biomarker expressions (from Panels C and D) are used in place of the CODEX-generated values in the kNN algorithm used to produce cell-type ground truth. An example predicted sample is shown. (**Panel F**) Using a deep learning model trained to predict phenotypic outcomes (Zheng et. al), the predicted cell types are used in place of the ground truth cell types to produce sample-level predictions for survival status, HPV status, and recurrence.

The *7-UP* framework consists of the following pipeline. We first select an optimal panel of seven biomarkers from the full CODEX biomarker panel. While the choice of which biomarkers to measure in a 7-plex imaging workflow can depend on clinician preference and disease subtype, we use a previously validated approach, concrete autoencoder (21), for automatically selecting informative biomarkers. This approach identified *DAPI*, *CD45RA*, *CD15*, *pan-cytokeratin* (*PanCK*), *HLA-DR*, *Ki67*, and *vimentin* (“main panel” in Table 1), which we use in our main experiments. We additionally report results using an alternative panel commonly used in immunology (4, 22) consisting of *DAPI*, *CD4*, *CD15*, *PanCK*, *CD8*, *Ki67*, and *vimentin* (“Alternative panel” in Table 1), and the results are consistent with the main panel. For comparison, we also report the performance of several panels containing completely distinct biomarkers (Table S1). Next, we extract cell-level spatial features across each of these seven biomarkers in the CODEX data set. To do this, we train a convolutional neural network (23) to learn spatial and morphological features from cell image patches generated from the full samples. We combine cell-level spatial features with average biomarker expression values to train a machine learning regression model (24) to impute the expression of the 30+ additional biomarkers (Fig. 1).

**Table 1.** Performance of 7-UP on the UPMC-HNC data set.

	Patchwise PCC	Patchwise *F*1
**UPMC-HNC data set**	**33 biomarkers**	**16 cell types**
7-UP model (seven biomarkers)	0.474 (0.006)	0.667 (0.002)
7-UP model (seven biomarkers + morphology) main panel	0.534 (0.009)	0.727 (0.002)
7-UP model (seven biomarkers + morphology) alternative panel	0.529 (0.007)	0.739 (0.002)

Biomarker imputation results are reported using the average patchwise PCC. Cell type predictions are reported using the patchwise weighted *F*1 score. The first row refers to the model trained without including morphological features in the input. The second and third rows refer to the models trained with morphological features. The main and alternative panels are described in the Materials and Methods section. Numbers in parentheses indicate the 95% bootstrapped confidence intervals.

To validate the veracity of the 7-UP imputed expressions, we use them to predict cell types and patient outcomes. We replace CODEX-measured expressions with the 7-UP imputed expressions in a k-nearest neighbor (kNN) algorithm used to determine cell type ground truth to generate cell type predictions. In turn, these predicted cell types are used as input in place of the CODEX-measured ground truth cell types in a graph neural network (GNN) (25) trained to produce sample-level predictions for patient-level survival status, HPV (human papillomavirus) status, and recurrence.

### Application of 7-UP to head and neck and colorectal cancer data sets

Our primary data set consists of 308 samples from 81 patients with head and neck squamous cell carcinomas at the University of Pittsburgh Medical Center (UPMC-HNC). Three external validation data sets are used: a head and neck squamous cell carcinomas data set with 38 samples from 11 patients from Stanford University (Stanford-HNC) to demonstrate generalization on the same disease, a colorectal cancer data set with 292 samples from 161 patients from Stanford University (Stanford-CRC) to demonstrate generalization to another disease, and a head and neck squamous cell carcinomas data set with 112 samples from 29 patients from Dana Farber Cancer Institute (DFCI-HNC) to demonstrate generalization to an additional clinical site. The number of samples, patients, coverslips, and total cells in each data set is described in Table S2. Phenotype annotations for UPMC-HNC are described in Table S3. UPMC-HNC is chosen as the primary training and evaluation data set as it contains the largest number of samples, coverslips, and total cells. We evaluate our models on held-out coverslips not seen during training to assess model robustness to technical artifacts across coverslips.

### Concordance of biomarker imputation

**Fig. 2.**
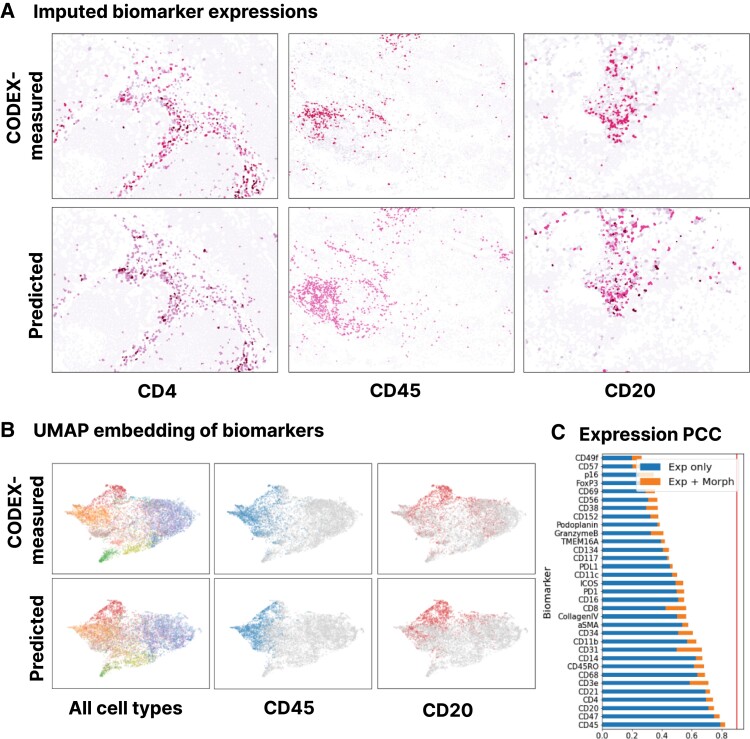
Biomarker imputation concordance on the UPMC-HNC data set. (**Panel A**) CODEX-measured versus predicted expressions for three biomarkers: CD4, CD20, and CD45. Samples shown have average patchwise PCC scores around the 50th percentile of all samples. (**Panel B**) A UMAP embedding was performed on the biomarkers of an equal sample of CODEX-measured and predicted cells. The first column is labeled by the ground truth cell types (legend from Fig. 1E); the second and third columns represent cells that express CD45 and CD20 (labeled by expressions greater than the 75th percentile CODEX-measured value). (**Panel C**) Patchwise PCC across all test samples for each biomarker. The “Exp only” bars represent the performance of a model trained only using average expression values as input; the “Exp + Morph” bars represent the performance of a model trained using both average expression values and morphology features.

*7-UP* achieves an average Pearson correlation coefficient (PCC) of 0.534 across all predicted biomarkers in the UPMC-HNC data set (Table 1). The predictive performance also holds across an alternative input panel (PCC of 0.529). Immune-related biomarkers like CD4, CD20, and CD45 are most accurately predicted, with PCCs above 0.70 (examples shown in Fig. 2).

### Predicting cell types from imputed biomarkers

We also measure the reliability of the imputed biomarkers by using them for determining cell types since cell type identification is a common task in analyses of CODEX data. Toward this task, *7-UP* achieves an *F*1 score of 0.727. The full CODEX-measured biomarker panel defines the ground truth labels in both models.

**Fig. 3.**
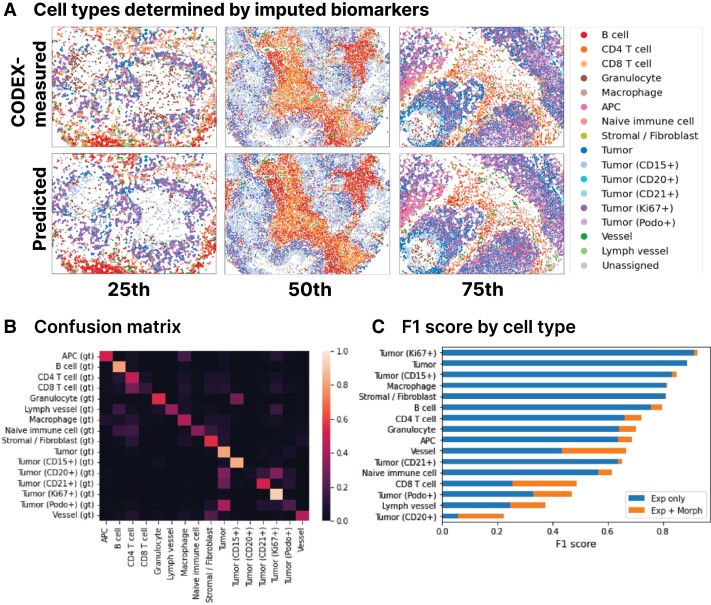
Cell type predictions closely match CODEX measurements (UPMC-HNC data set). (**Panel A**) CODEX-measured and predicted cell types are shown side-by-side on 25th, 50th, and 75th percentile samples (by patchwise *F*1 score). (**Panel B**) Left: confusion matrix between the kNN-determined ground truth cell types (rows) and ML imputed cell type (columns). Right: breakdown of patchwise *F*1 score by cell type. The “Exp only” bars represent the performance of a model trained using only average expression values, and the “Exp + Morph” bars represent the performance of a model trained using both average expression values and morphological features.

We examine how accurately the predicted cell types retain local cell neighborhood structures by comparing the spatial adjacency matrices (Fig. S1). These were produced by projecting the cells into a graph representation described in Zheng et al. (25) and then counting the relative frequencies of spatially adjacent cells. Comparing the two matrices shows that local clusters of cell types are well preserved (root mean square distance of 0.0357). We additionally verify that the predicted cell types closely match the true distribution by projecting the predicted and CODEX-measured biomarker expressions using UMAP (Uniform Manifold Approximation and Projection) (Fig. 2B) and visualizing the cell type labels (Fig. 3).

### Predicting patient phenotypes from predicted cell types

**Fig. 4.**
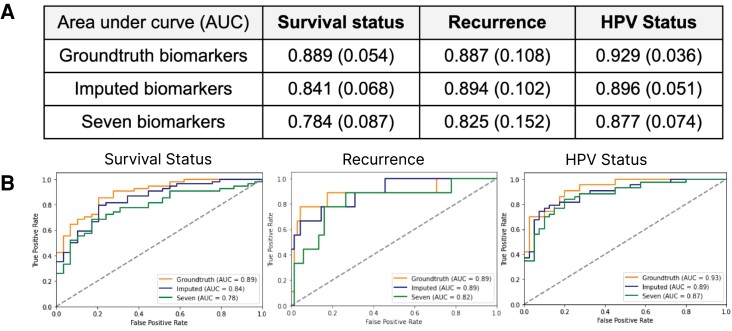
Imputed biomarkers are useful for predicting patient phenotypes (UPMC-HNC data set). For reference, the performance of a model trained to predict phenotypes using only the seven input biomarker panel was reported as well (“seven biomarkers”). (**Panel A**) Three phenotypic outcomes using imputed vs. CODEX-measured vs. seven biomarkers. AUC score reported (95% bootstrapped confidence interval reported in parentheses). (**Panel B**) ROC curves of three phenotypic outcomes.

To validate the reliability of the cell types determined by *7-UP*-imputed biomarkers, we use them to predict three patient phenotypic outcomes: HPV infection status, primary outcome (survival), and recurrence of disease. This is to demonstrate the usefulness of the predicted cell types beyond the mean *F*1 score by showing that cell type prediction using imputed biomarkers can be robustly used for downstream tasks like survival prediction. To this end, we use a graph-based deep learning model (25) trained using ground truth cell types from the UPMC-HNC data set to predict these three binary outcomes. To evaluate the veracity of our predicted cell types, we replace the CODEX-measured cell types used to make the baseline prediction with the predicted cell types as input to the model. The results demonstrate that the imputed cell types can predict phenotypic outcomes at a level comparable to the ground truth labels (Fig. 4).

### Cross-site and cross-disease generalization

**Fig. 5.**
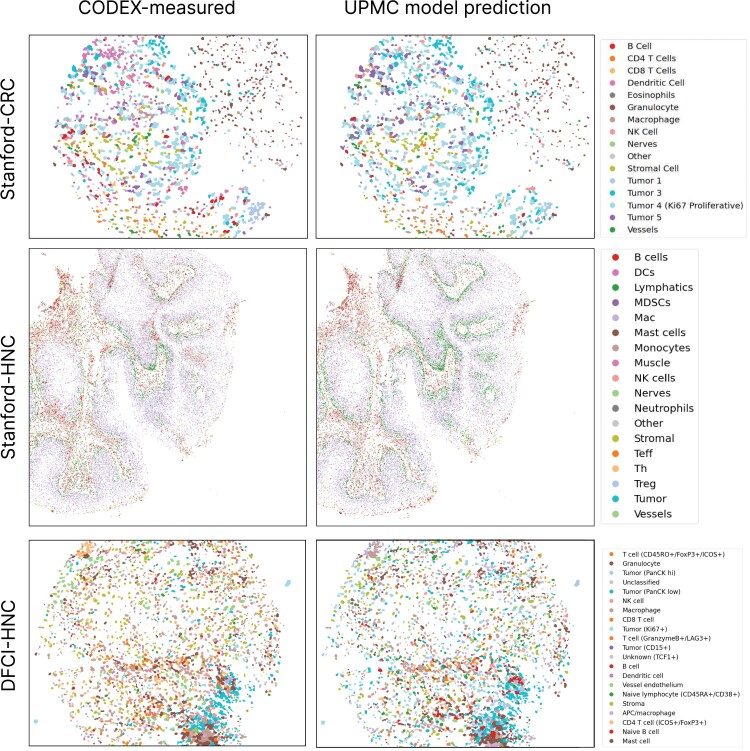
Visualization of CODEX-measured and UPMC-HNC model-predicted cell types on three validation data sets. Fiftieth percentile (by *F*1 score) samples are shown, labeled by cell types.

Finally, we evaluate our model on two head and neck cancer data sets from (Stanford-HNC) and DFCI (DFCI-HNC) and a colorectal cancer data set (Stanford-CRC). The biomarker imputation and cell type prediction performances remain stable (e.g. for Stanford-CRC: 0.489 vs. 0.583 PCC and 0.614 vs. 0.605 *F*1) even when evaluated on a different clinical site and cancer type (Table 2 and Fig. 5), indicating that the model's performance is robust when evaluated on unseen data. For each cross-site evaluation, a breakdown of PCC per biomarker is visualized in Fig. S2.

**Table 2.** 7-UP generalizes well to other data sites and disease types.

	Patchwise PCC	Patchwise *F*1
**Stanford-CRC data set**	**24 biomarkers**	**16 cell types**
with UPMC-HNC model	0.489 (0.024)	0.614 (0.004)
with Stanford-CRC model	0.583 (0.031)	0.605 (0.004)
**Stanford-HNC data set**	**26 biomarkers**	**18 cell types**
with UPMC-HNC model	0.475 (0.005)	0.757 (0.001)
With Stanford-HNC model	0.545 (0.004)	0.773 (0.001)
**DFCI-HNC data set**	**20 biomarkers**	**21 cell types**
with UPMC-HNC model	0.394 (0.016)	0.545 (0.006)
With DFCI-HNC model	0.531 (0.021)	0.590 (0.004)

Imputed biomarker and predicted cell type performance are demonstrated on three external validation data sets (Stanford-CRC, Stanford-HNC, and DFCI-HNC). The UPMC-HNC model's performance is reported on each data set, along with a reference model that has been trained on the validation data set. Metrics are reported with 95% confidence intervals in parentheses.

### Training on only one coverslip

Because highly multiplexed fluorescence imaging platforms like CODEX are more resource-intensive than standard fluorescence immunochemistry imaging, one might wish to image only one coverslip with CODEX and then train a model to impute additional biomarkers on other coverslips imaged with a 7-plex system. We experiment with only training the imputation model on one coverslip (24% of the entire training data) and report that performance from this model retains the same cell type prediction performance (Table S4A). Even in this low data regime, the model can still robustly impute cell types without sacrificing performance.

### 7-UP leverages cell morphology

Finally, we verify that the deep learning model learns morphology features useful for imputing biomarkers for single cells beyond the mean expression values. Using only average cell expression values as input features, our method achieves an average PCC of 0.474 across all predicted biomarkers in the UPMC-HNC data set. When adding additional morphology features, the performance improves to 0.534 PCC (Table 1). Similarly, when determining cell types, a model which uses only the average expressions of seven biomarkers achieves an average patchwise-weighted *F*1 score of 0.667. In contrast, the model with biomarkers imputed using morphology features achieves an *F*1 score of 0.727.

Additionally, to demonstrate the usefulness of the context channels used in the deep learning model, we performed an ablation experiment (Table S4B) where we evaluated a model trained using the context channels and a model trained without (only using the single-cell image). We observe an improvement (0.016 PCC and 0.014 *F*1) with the inclusion of context channels, indicating that features from the cell's neighborhood are useful in determining information about the cell.

As an example, classification performance on vessel cells increases from 0.44 to 0.67 *F*1 score when including morphological features. Of the vessel cells incorrectly classified without morphology, 70% were predicted as stromal cells. Figure S3 visualizes examples of cells that were corrected with the inclusion of morphology. Though vessel cells and stromal cells share a similar protein expression composition (vimentin, aSMA, CollagenIV, and CD47), vessel cells uniquely express CD31 and CD34. Indeed, the model with morphology more accurately predicts CD31 (PCC: 0.561 vs. 0.416) and CD34 (PCC: 0.586 vs. 0.429). We can infer that the model was able to better predict the expression of these two biomarkers with morphological information of the seven biomarkers than with only average expression.

## Discussion

A promise of spatial proteomics is to exploit the rich spatial information present in TME images, which enables higher-dimensional analyses beyond bulk or average protein expressions. The capability to infer biomarker co-expression patterns from cell morphologies and spatial structures of cell niches in the TME can enable CODEX-like insights from a smaller panel of biomarkers.

High-plex immunofluorescence (IF) techniques like CODEX enable an unprecedented understanding of TME and tissue architecture but have seen limited clinical (diagnostic or prognostic) utility due to their cost and data generation times. On the other hand, standard IF or immunostaining workflows, which image between one and seven biomarkers, are widely available. 7-plex mIF panels are becoming more common in clinical settings. Our proposed method aims to unlock the richer TME representations available with CODEX by upleveling existing 7-plex data through learning biomarker co-expression and morphological patterns.

Even a small subset of biomarkers may contain sufficient signal to reconstruct a much larger subset of biomarkers: for instance, some biomarkers regularly co-express with other biomarkers (e.g. CD20 and CD21 in B cells), while others can be inferred from the cell's morphology (e.g. the nucleus and cytokeratin expression of a proliferating tumor cell may indicate Ki67 expression). Indeed, our results suggest that learning these relationships is useful and that the imputed biomarker expressions are reliable enough to be used in place of CODEX-measured expressions for the primary tasks of resolving cell types and predicting phenotypic outcomes.

The panel selection procedure in Fig. 1A demonstrates one method for selecting input biomarkers, which does so by maximizing the average reconstruction accuracy across all other CODEX-measured biomarkers. In scenarios where multiplex imaging data have been previously imaged and collected, *7-UP* can be deployed directly on the predefined subset of biomarkers, thus removing the need for panel selection.

The ability to determine a subset of biomarkers in silico (i) gives users immediate access to a larger set of biomarkers beyond what has been experimentally measured and (ii) frees up resources to measure more novel and biologically relevant biomarkers. Thus, in addition to upleveling 7-plex systems, *7-UP* can also push CODEX systems from ∼40 biomarker measurements to 60 or more, enabling even greater cell type differentiation and disease characterization.

### Limitations

While some biomarkers are imputable with a high degree of confidence, others are not as easily predicted. This is a consequence of the inherent limitations of a 7-plex panel. Intuitively, increasing the panel beyond seven biomarkers would increase the number of strongly predicted biomarkers but would also surpass the technical limitation of most clinical multiplex workstations. Additionally, since biomarkers are differentially expressed based on their unique TME, training in a variety of disease contexts is key to ensuring generalizability. Picking an informative panel of biomarkers is also an important decision and ought to reflect the nature of the disease and TME that one wishes to understand.

Studies using IF data pose challenges in terms of data validation, noise sources, and interpretative analysis, like cell typing. Specific challenges with IF and other tissue stains include the difficulty of cell segmentation; the presence of tissue artifacts such as folds, tears, and distortions; and image processing issues such as the alignment of tiles or channels. We recognize that CODEX and other highly multiplexed IF assays are not exempt from these challenges (19).

Our aim in evaluating our model across diverse samples from held-out patients, clinical sites, and disease types is in part to increase confidence in the underlying consistency in the CODEX measurements present across data sets. However, we recognize that measurement errors may still be present in mIF data even after strict quality control (QC) measures. Despite these systemic limitations, we highlight *7-UP*'s performance robustness through two additional points. First, though error-free cell segmentation is an open problem, our manuscript employs a state-of-the-art deep learning approach (DeepCell (26)) in order to achieve high-performing results. Although segmentation was used to train and test the model, we note that an advantage of *7-UP* is that it could easily be adapted to work with segmentation-free images. Second, since our data were processed in an internally consistent manner—i.e. the pipeline for acquiring test data and training data was identical—mIF data accuracy should not affect the 7-UP model's ability to generalize. Furthermore, we demonstrate that the model is still able to learn biologically relevant information as reflected through cell type and patient phenotype predictions.

## Materials and methods

### CODEX data collection

All samples are prepared, stained, and acquired following CODEX User Manual Rev C (https://www.akoyabio.com).

#### Coverslip preparation

Coverslips are coated with 0.1% poly-L-lysine solution to enhance adherence of tissue sections prior to mounting. The prepared coverslips are washed and stored according to the guidelines in the CODEX User Manual.

#### Tissue sectioning

Formaldehyde-fixed paraffin-embedded (FFPE) samples are sectioned at a thickness of 3–5 *µ*m on the poly-L-lysine-coated glass coverslips.

#### Antibody conjugation

Custom conjugated antibodies are prepared using the CODEX Conjugation Kit, which includes the following steps: (i) the antibody is partially reduced to expose thiol ends of the antibody heavy chains, (ii) the reduced antibody is conjugated with a CODEX barcode, (iii) the conjugated antibody is purified, and (iv) antibody storage solution is added for antibody stabilization for long-term storage. Postconjugated antibodies are validated by SDS–polyacrylamide gel electrophoresis (SDS–PAGE) and QC tissue testing, where IF images are stained and acquired following standard CODEX protocols, and then evaluated by immunologists.

#### Staining

CODEX mIF imaging was performed on FFPE patient biopsies using the Akoya Biosciences PhenoCycler platform (also known as CODEX). Five-micrometer-thick sections were mounted onto poly-L-lysine-treated glass coverslips as tumor microarrays. Samples were pretreated by heating on a 55 °C hot plate for 25 min and cooled for 5 min. Each coverslip was hydrated using an ethanol series: two washes in HistoChoice Clearing Agent; two in 100% ethanol; one wash each in 90%, 70%, 50%, and 30% ethanol solutions; and two washes in deionized water (ddH2O). Next, antigen retrieval was performed by immersing coverslips in Tris-EDTA pH 9.0 and incubating them in a pressure cooker for 20 min on the high setting, followed by 7 min to cool. Coverslips were washed twice for 2 min each in ddH2O and then washed in Hydration Buffer (Akoya Biosciences) twice for 2 min each. Next, coverslips were equilibrated in Staining Buffer (Akoya Biosciences) for 30 min. The conjugated antibody cocktail solution in Staining Buffer was added to coverslips in a humidity chamber and incubated for 3 h at room temperature or 16 h at 4°C. After incubation, the sample coverslips are washed and fixed following the CODEX User Manual.

#### Data acquisition

Sample coverslips are mounted on a microscope stage. Images are acquired using a Keyence microscope that is configured to the PhenoCycler Instrument at a 20× objective. All of the sample collections were approved by institutional review boards.

To correct for possible autofluorescence, “blank” images were acquired in each microscope channel during the first cycle of CODEX and during the last. For these images, no fluorophores were added to the tissue. These images were used for background subtraction. Typically, autofluorescence will decrease over the course of a CODEX experiment (due to repeated exposures). Thus, to correct each cycle, our method determines the extent of subtraction needed by interpolating between the first and last “blank” images.

#### Quality control

For each study, biomarker staining quality was evaluated by an immunologist for specificity and signal-to-noise characteristics. In Fig. S4, we have included examples of core quality reviews from the UPMC-HNC data set. Each biomarker was scored for staining performance using the following criteria: 3, highest quality with cell-type and subcellular specificity, low background signal; 2, good quality with cell-type and subcellular specificity, possibly some background signal; 1, some cell-type and subcellular specificity may be detected, background signal may be high; 0, no biomarker specificity observed, high background signal; ND, not determined, stain specificity could not be determined. For the UPMC-HNC study, for instance, 41 biomarkers in the panel were scored: 17 biomarkers had a score of 3, 9 biomarkers had a score of 2, 13 biomarkers had a score of 1, 1 biomarker had a score of 0, and 1 biomarker had a score of ND (not determined). Markers with poor quality were removed from the study prior to analysis. The vast majority of markers received a passing grade in the analysis (for UPMC-HNC, 39/41 or 95.1%). Representative images for all markers have been added to the study as an interactive HTML report (report link; download the HTML file and open in a browser). Representative grades have been added to the manuscript as a figure as well (Fig. S5). The analyses and procedures performed in this report are repeated across the other studies used in this manuscript.

Additionally, we have included a visualization of cell types with relevant biomarkers on three samples from the UPMC-HNC study (Fig. S6). Finally, we visualize the average biomarker expressions by cell type for each study that was evaluated (Fig. S7).

#### Data sets

The UPMC-HNC and Stanford-HNC data sets have one held-out coverslip for model validation and one held-out coverslip for model evaluation. The Stanford-CRC data set has half of one coverslip randomly split and held out for model validation and one held out for model evaluation. The DFCI-HNC data set has one coverslip randomly split by patients for model evaluation.

### Choice of input biomarkers

Our 7-UP framework can be applied to any set of input biomarkers, though the imputation performance improves if the input markers are particularly informative. Concrete autoencoder (21) is an unsupervised neural network that determines the subset of biomarkers that are most useful for reconstructing the entire CODEX panel (Fig. 1A). The concrete autoencoder takes a full set of input biomarker expressions and outputs a feature importance score for each biomarker. This approach achieves very similar results when compared to a naïve greedy algorithm (iteratively including the most important biomarkers in the model) but is more computationally efficient.

### Biomarker expression preprocessing

Single-cell expression was computed for each biomarker by (i) applying a deep learning cell segmentation algorithm (DeepCell) (26) on the DAPI biomarker channel (nuclear stain) to obtain nuclear segmentation masks, (ii) successively dilating segmentation masks by flipping pixels each time with a probability equal to the fraction of positive neighboring pixels (repeated nine times), (iii) computing the mean expression value across pixels within the single cell, and (iv) normalizing the expression values across all cells in a sample using quantile normalization and arcsinh transformation followed by a *z*-score normalization:


$$z\mathrm{score}\left(\mathrm{arcsinh}\left(\frac{x}{5q_{0.2}(x)}\right)\right),$$

where zscore is defined given *μ* and *σ*, the mean and standard deviation across all cell expression values in the sample:


$$z\mathrm{score}(x)\;\;=\;\;\frac{x-\mu }{\sigma },$$

where *x* is the vector of a biomarker’s values in a sample, *arcsinh* is the inverse hyperbolic sine function; and $q_{0.2}(x)$ is the 20th percentile of *x*.

### Image patch generation

After preprocessing (tile and cycle alignment, stitching, deconvolution, and background correction), CODEX data are available as multichannel OME-TIFF files, with each image channel corresponding to the fluorescence signal (expression) of a distinct biomarker probe. To prepare the input image patches for the deep learning model, we perform the following: all pixel values for a biomarker in a sample are normalized using ImageJ's AutoAdjust function. An image patch (224 px × 224 px) is then generated for each cell, for each biomarker, in the region. Each patch consists of three channels. The first channel contains the segmented cell only, rescaled 3×, centered with zero padding around the cell; the second channel contains a crop of the neighborhood (∼20 cells) centered around the cell (∼84 *µ*m) at 1× scaling, and the third channel contains a crop of the neighborhood (∼80 cells) at 0.5× scaling also centered around the cell (∼42 *µ*m). These channels are visualized in Fig. 1B. As a reference, all coverslips are imaged at a resolution of 0.3775 *µ*m per pixel.

### Deep learning model

We trained a ResNet-50 (23) deep learning model to learn cell shape features as well as spatial information of cell neighborhoods. We find that training the model on cell type classification enables it to learn an effective morphology featurizer. We start with a model with weights pretrained on the ImageNet data set (27). The model takes as input a 224 × 224 × 21 size tensor, where the 21 channels correspond to stacking 7 input biomarkers with 3 feature channels each. The last layer is modified to classify over one-hot encoded cell types. The model is trained with categorical cross-entropy loss, and a cell-wise *F*1 score is computed at each validation step. The learning rate is initialized at 1e−4, and decays by a factor of 0.2 if the validation *F*1 score does not improve over 5,000 steps. Training stops after 75,000 steps of no improvement, and the model with the highest validation *F*1 score is chosen. To improve model robustness, we trained an ensemble of five identical models with different random weight initializations and computed the mean prediction across all models to obtain a final model score. While each individual model's performance is comparable, we find that aggregating the predictions boosts overall performance (Fig. S8). All models were implemented and trained using PyTorch (28), a Python deep learning framework. To gain insights into what is learned by the deep learning model, we extracted morphology features (using the HistomicsTK Python library) from the cell segmentation masks and correlated them with the top 10 principal components (PCs) of the deep learning model-extracted last layer embeddings (Fig. S9). Earlier PCs are more correlated with cell size features like area and axis length while later PCs (i.e. five) are more correlated with cell shape features like eccentricity and axis ratio. While the manual features correlate well with the deep learning embedding features, interpretation of our deep learning model is inherently limited given that the morphology features are extracted from the cell segmentation mask, while the input patch to the deep learning model includes the cell's context.

### Biomarker imputation model

XGBoost (24), a gradient-boosting decision tree algorithm shown to achieve top performance in tabular data regression, is used for imputing single-cell biomarker expressions. The model takes as input the cell expression values of the seven input biomarkers, along with, in the case of adding morphology information, a probability vector corresponding to cell type predictions from the deep learning model. It is then trained to jointly predict the expression values of the remaining biomarkers. We find that directly using the output probabilities improves model performance more than using the final featurization layer. We used squared error loss, a learning rate of 0.1, 500 estimators, a max depth of 3, a per-tree column sampling of 0.7, and GPU (Graphics Processing Unit)-accelerated training. All other hyperparameters are default settings in the XGBoost Python library.

### Dimensionality reduction

To visualize the concordance of CODEX-measured and imputed biomarkers, we randomly sample 10,000 cells with CODEX-measured biomarker values and 10,000 cells with imputed biomarker values and fit a UMAP (29) dimensionality reduction model on the combined set. We then plot the projected 2D data points separately and color them by ground truth cell types, expression of CD45, and expression of CD20 (the latter two use the 75th percentile expression value as a binary threshold). The UMAP model is trained using the RAPIDS.ai GPU-accelerated implementation with default settings.

### Patchwise metrics

Given the naturally high degree of intercellular expression variation within local neighborhoods of cells, we report biomarker and cell type predictions aggregated within a local cell neighborhood. Figure S10 shows the relationship between the choice of cell neighborhood patch size (in pixels) and the average PCC and *F*1 score. The patchwise PCC of a biomarker is computed as the PCC between the CODEX-measured and imputed patchwise average expressions. Patchwise *F*1 is computed by considering a patch as positive if at least one cell is assigned to that cell type and then calculating the *F*1 score across patches.

### Cell type ground truth and predictions

To produce cell type labels, we first obtained a cells-by-features biomarker expression matrix—for each marker, we took the average signal across all pixels in a segmented cell. This matrix was normalized and scaled as described above, and then PC analysis was performed. We constructed a nearest neighbor graph (*k* = 30) of cell expression in PC space with the top 20 PCs and then performed self-supervised graph clustering (30) on the result. Clusters were manually annotated according to their cell biomarker expression patterns. This procedure was performed on a subset of 10,000 cells and subsequently used to train a kNN algorithm. This algorithm was used to transfer labels to the entire data set.

In our experiments where we generate cell type predictions based on the imputed biomarkers, we use this trained kNN algorithm and substitute the subset of expressions for which we are imputing with the imputed values from the ML model. Thus, for the UPMC-HNC data set with 41 total biomarkers, 7 biomarkers will be the CODEX-measured values, and 33 biomarkers will be imputed.

### Survival outcome prediction

Additionally, three phenotypic patient outcomes from the UPMC-HNC data set are evaluated: survival status [No Evidence of Disease (NED) versus Died of Disease (DOD)], HPV status (a significant indicator of cancer prognosis), and recurrence (if the cancer recurs within 5 years after diagnosis).

We used a GNN-based model (25) trained on using cell types to predict patient phenotypic outcomes. This model transforms the structure of each sample into a graph network, where cells are connected by edges to neighboring cells. It then pools information about the neighboring cells’ cell types to output an outcome probability score for each cell. The sample predictions are generated by averaging the scores across all cells in that sample. We evaluated models that have been trained on three patient phenotypic outcomes: survival status, HPV status, and recurrence. To validate the utility of our imputed cell types, we replace the original annotated cell type labels with the predicted cell types produced from the imputed biomarkers. The results are reported in Fig. 4, where we see that performance on these three tasks is comparable between using the imputed biomarkers and the CODEX-generated biomarkers.

## Supplementary Material

pgad171_Supplementary_Data

## Data Availability

All codes used to produce the results in this paper are available at https://gitlab.com/enable-medicine-public/7-up. All data are included in the manuscript and/or supporting information. This manuscript was posted on a preprint: https://doi.org/10.1101/2022.06.03.494624.
