# Supplementary material for: 7-UP: Generating in silico CODEX from a small set of immunofluorescence markers
Source: PNAS Nexus. 2023 May 19;2(6):pgad171. doi: 10.1093/pnasnexus/pgad171 (PMC10236358; doi:10.1093/pnasnexus/pgad171)
Supplement: pgad171_Supplementary_Data [file pgad171_supplementary_data.pdf]

# Supplemental Tables and Figures

| **Panel of 7 biomarkers (UPMC-HNC)** | **Single-cell PCC** |
|---|---|
| DAPI, CD57, CD49f, CD38, TMEM16A, CD69, p16 | 0.26 |
| DAPI, CD49f, p16, CD16, CD69, CD38, CD152 | 0.28 |
| DAPI, CD16, CD56, CD11c, CD134, p16, CD11b | 0.35 |
| DAPI, CD45RA, CD15, PanCK, HLA-DR, Ki67, Vimentin **(Main panel)** | 0.36 |
| DAPI, CD4, CD15, PanCK, CD8, Ki67, Vimentin **(Alternative panel)** | 0.38 |

**Table S1:** Choice of input biomarkers on the performance of 7-UP. Three varying biomarker panels along with the main and alternative panels presented in Table 1 and the corresponding average single-cell PCC of the CODEX-measured and predicted biomarker expressions. For each row, the panel of seven biomarkers is used to predict the rest of the hold-out thirty-three biomarker expressions.

| Dataset | No. of samples | No. of patients | No. of coverslips | No. of total cells |
|---|---|---|---|---|
| UPMC-HNC | 308 | 81 | 8 | 2,061,102 |
| Stanford-HNC | 38 | 11 | 6 | 1,643,491 |
| Stanford-CRC | 292 | 161 | 4 | 632,280 |
| DFCI-HNC | 112 | 29 | 1 | 259,620 |

**Table S2:** Descriptions of the four CODEX datasets used for training and evaluating 7-UP.

## A. Phenotype annotations for UPMC-HNC dataset.

| Dataset | $N_{sample}$ | $N_{patient}$ | $N_{batch}$ | $N_{cell}$ | Phenotype Annotations | | | | |
|---|---|---|---|---|---|---|---|---|---|
| | | | | | Primary Outcome | Survival Length | Recurrence | Recurrence Interval | Other |
| UPMC-HNC | 308 | 81 | 7 | 2061102 | no evidence of disease (NED): **197**<br>non-NED: **111** | 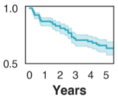 | recurred: **31**<br>not recurred: **253** | N/A | HPV infected: **158**<br>not HPV infected: **150** |



## B. Description of the primary site of tumor for UPMC-HNC dataset.

| Primary site of tumor (UPMC-HNC dataset) | No. of samples |
|---|---|
| Tonsil | 129 |
| Supraglottic | 61 |
| Base of Tongue | 52 |
| Larynx | 20 |
| Hypopharynx | 19 |
| Pyriform Sinus | 9 |
| Glottic | 9 |
| Oropharynx | 6 |
| Mouth (retromolar area) | 3 |

**Table S3: A:** Description of phenotype annotations for UPMC-HNC. **B:** Counts of the primary site of tumor across 308 samples in the UPMC-HNC dataset.

| A. One coverslip | **Patchwise PCC** | **Patchwise F1** |
|---|---|---|
| **UPMC-HNC** | **33 biomarkers** | **16 cell types** |
| All training coverslips | 0.523 (0.006) | 0.723 (0.002) |
| One coverslip (~24% of training data) | 0.493 (0.005) | 0.715 (0.002) |

| B. Context | **Patchwise PCC** | **Patchwise F1** |
|---|---|---|
| **UPMC-HNC** | **33 biomarkers** | **16 cell types** |
| Reference with context | 0.523 (0.006) | 0.723 (0.002) |
| Without context | 0.507 (0.005) | 0.709 (0.002) |

**Table S4:** 7-UP ablation studies. In Table A, the 7-UP model is trained using only data coming from one coverslip, which represents about a quarter of the total training data. In both experiments, the ablated model performs comparatively similar to the reference model. In Table B, the 7-UP model is trained with only the “Cell only” channel as described in Figure 1b, thus discarding the two context channels. The performance of this model is reported in the second row (“Without context”), and compared to a model trained with all three channels (“Reference with context”).

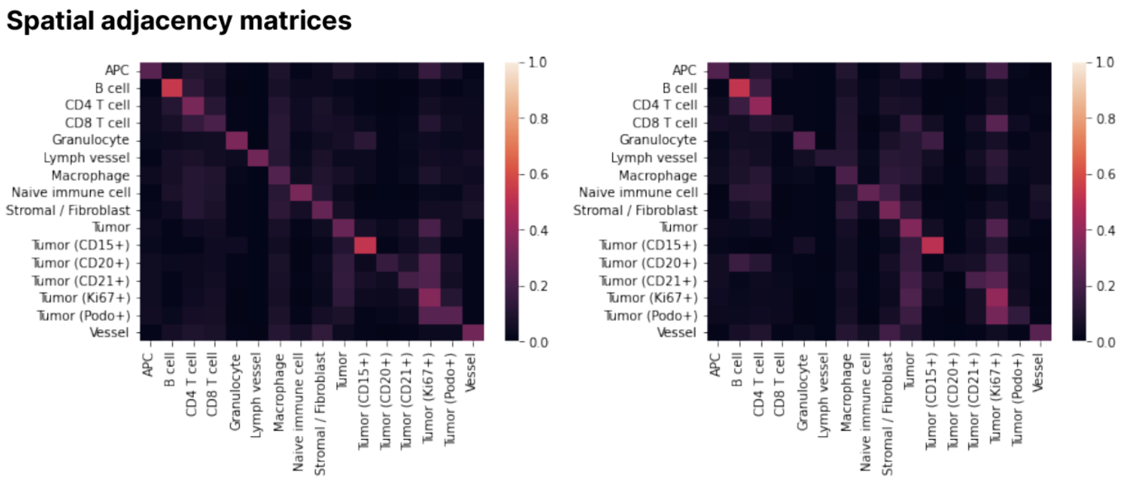


**Figure S1**: Spatial adjacency matrix agreement. To assess how well the cell type predictions preserve local neighborhood structures, we consider spatial adjacency matrices, which show the row-normalized counts of cell-cell neighbors within the UPMC-HNC test set.

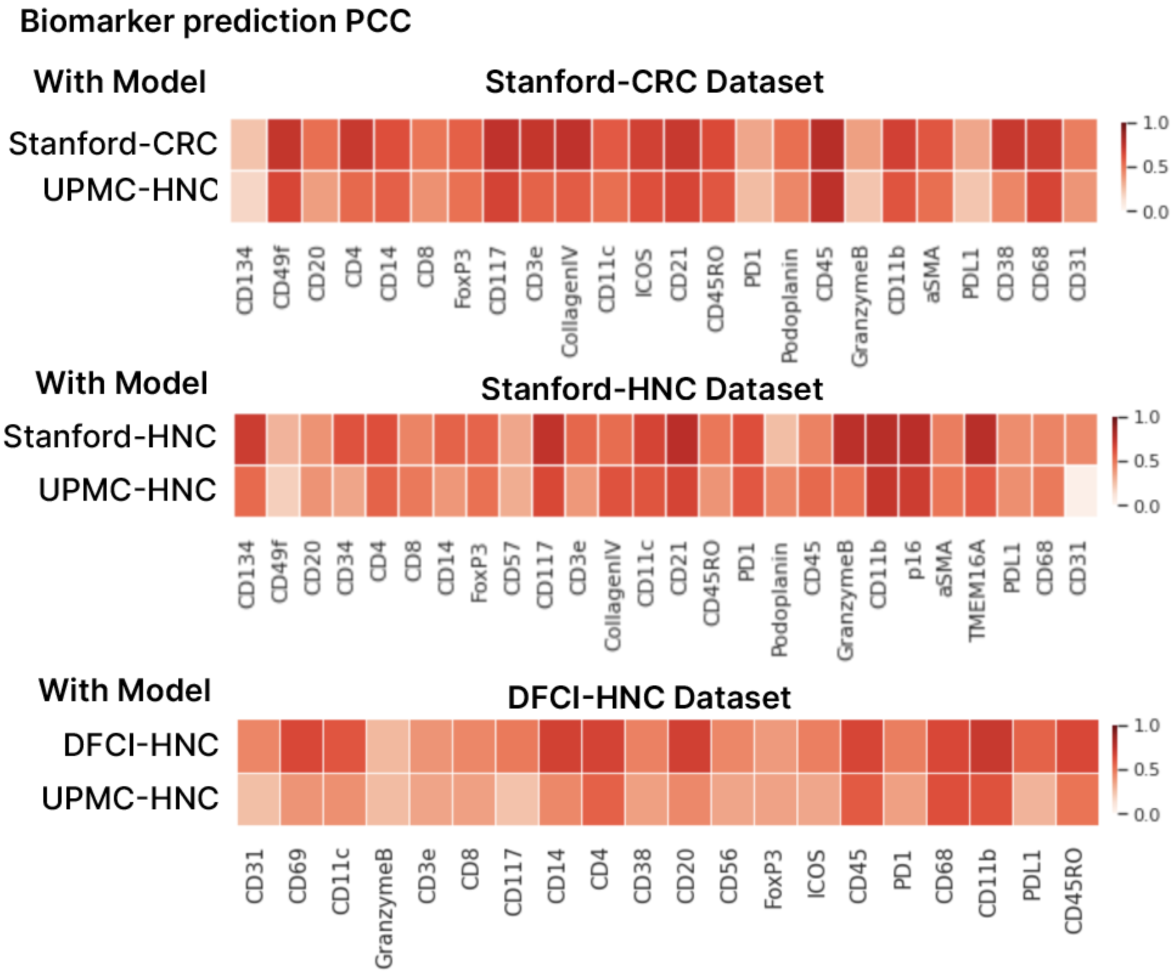


**Figure S2:** A breakdown of patchwise PCC per biomarker is visualized for each cross-site evaluation.

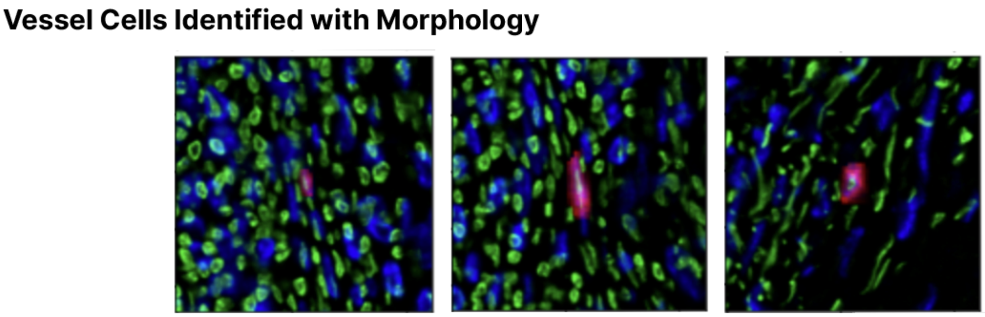


**Figure S3:** Vessel cells identified with morphology. Three patches of vessel cells were incorrectly classified as stromal cells but correctly classified with the inclusion of spatial information. In each patch, the DAPI stain is shown in three spatial scales: the cell morphology is presented in red, the 1x resolution context around the cell is shown in blue, and the 0.5x resolution context around the cell is shown in green.

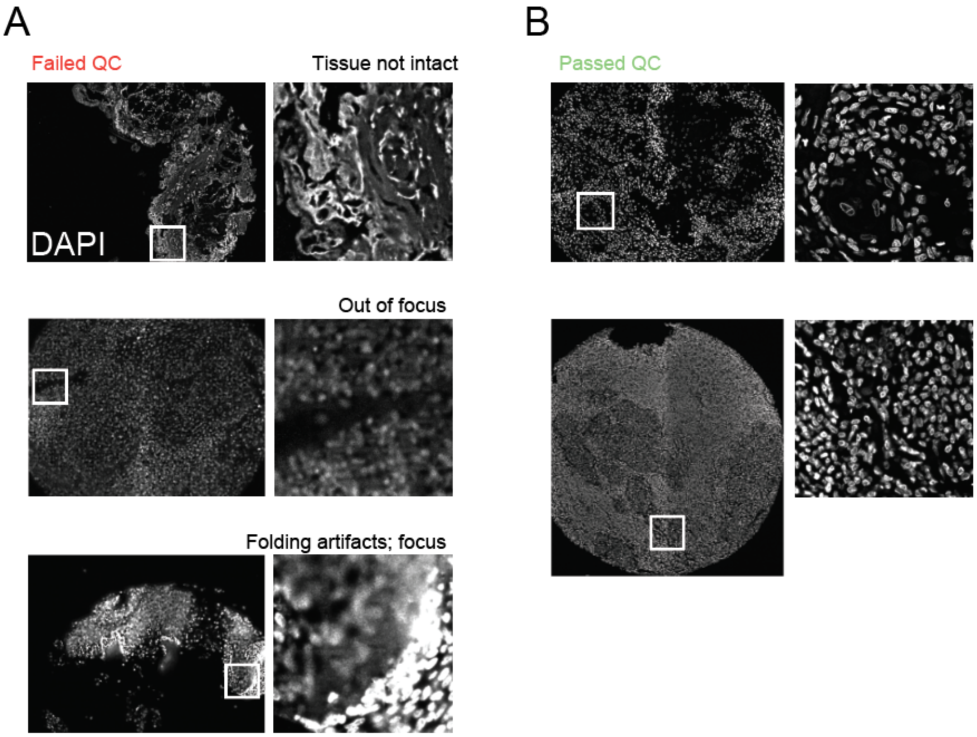


**Figure S4:** Examples of core quality reviews from the UPMC-HNC dataset. (A) Tissue cores were rejected from the analysis due to tissue quality, focus issues, or tissue artifacts. The reason for failure is given in each case. (B) Examples of cores that were kept in the analysis.

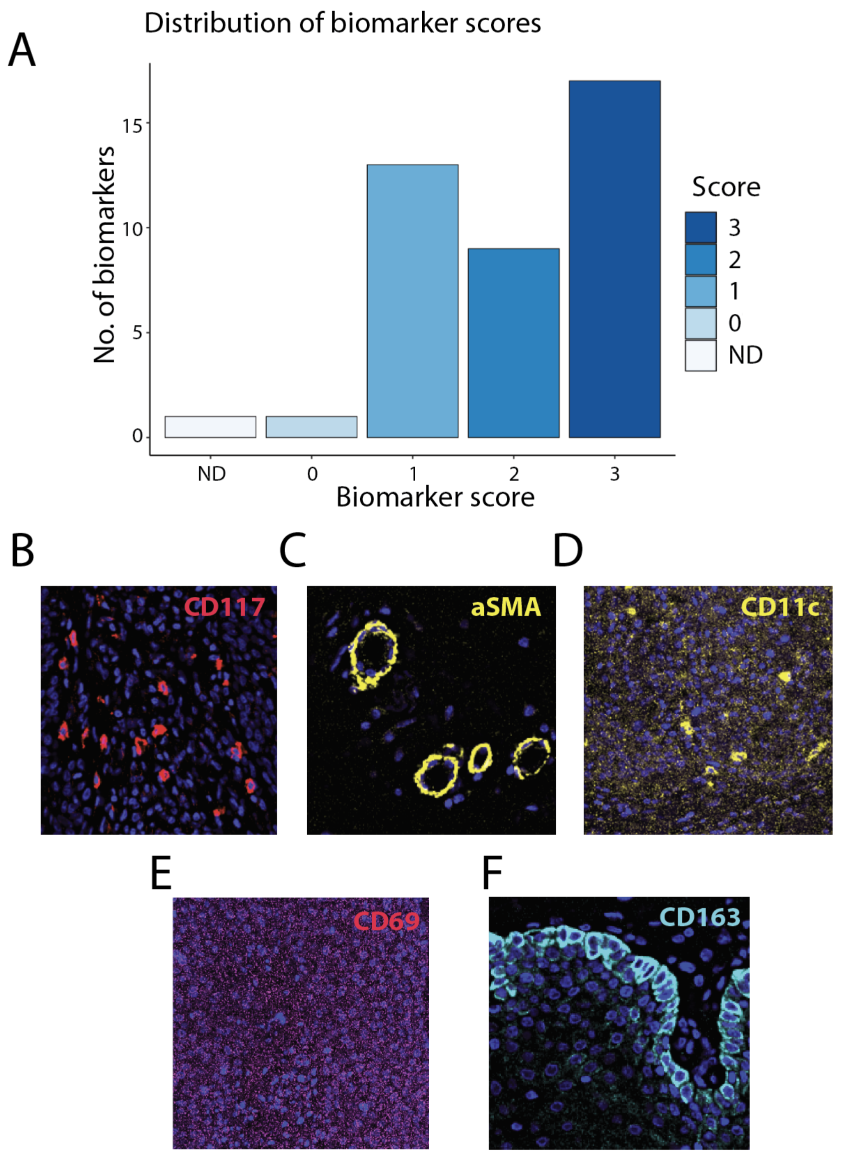


**Figure S5:** Examples of biomarker stain quality review from the UPMC-HNC dataset. (A) A summary of biomarker grades assigned after a manual review of randomly selected acquisitions. All biomarkers with grade 1 or above are considered sufficient quality for downstream analysis. (B-F) Examples of markers with scores of 3, 2, 1, 0, and ND, respectively. Scores are defined in the Methods section.

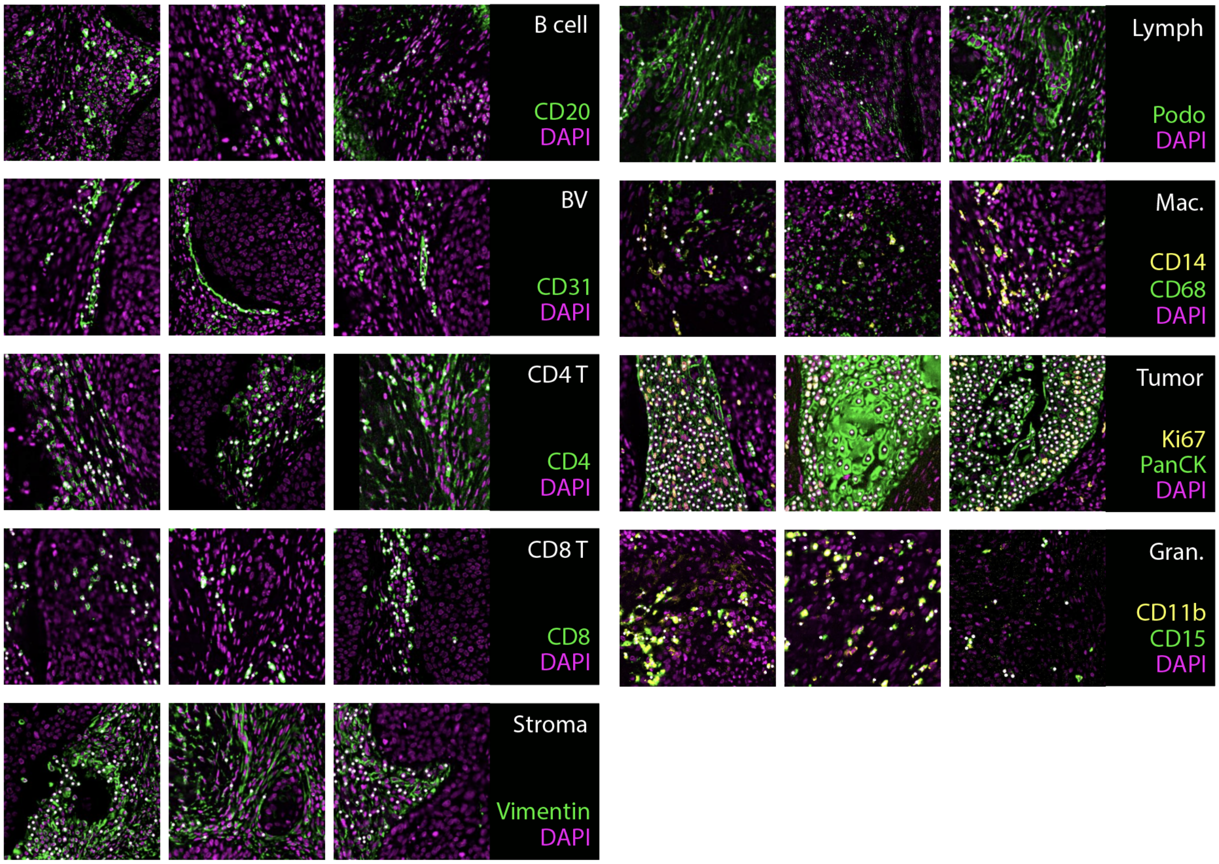


**Figure S6:** Visualization of cell types (white dots) presented alongside key representative biomarkers (green) and DAPI stain (purple). Each of the nine sections contains three patches from a different sample obtained from the UPMC-HNC dataset. Each of the cell types is consistently co-located with their corresponding biomarkers.

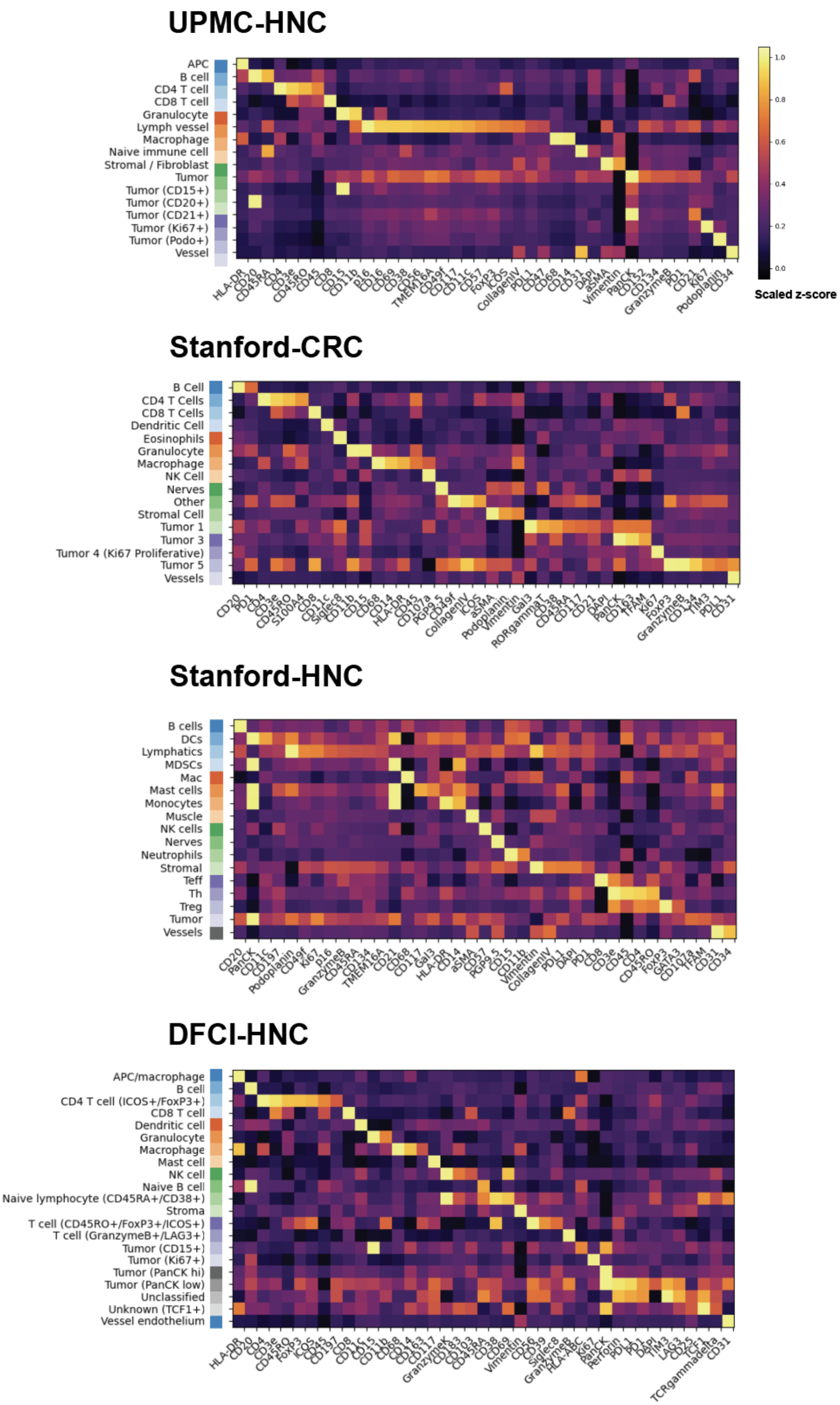
UPMC-HNC
APC
B cell
CD4 T cell
CD8 T cell
Granulocyte
Lymph vessel
Macrophage
Naive immune cell
Stromal / Fibroblast
Tumor
Tumor (CD15+)
Tumor (CD20+)
Tumor (CD21+)
Tumor (Ki67+)
Tumor (Podo+)
Vessel
HLA-DR CD20 CD45RA CD4 CD3e CD45RO CD45 CD8 CD15 CD11b p16 CD16 CD69 CD38 CD56 TMEM16A CD49f CD117 CD11c CD57 FoxP3 ICOS CollagenIV PDL1 CD47 CD68 CD14 CD31 DAPI aSMA Vimentin PanCK CD152 CD134 GranzymeB PD1 CD21 Ki67 Podoplanin CD34
1.0
0.8
0.6
0.4
0.2
0.0
Scaled z-score
Stanford-CRC
B Cell
CD4 T Cells
CD8 T Cells
Dendritic Cell
Eosinophils
Granulocyte
Macrophage
NK Cell
Nerves
Other
Stromal Cell
Tumor 1
Tumor 3
Tumor 4 (Ki67 Proliferative)
Tumor 5
Vessels
CD20 PD1 CD4 CD3e CD45RO S100A4 CD8 CD11c Siglec8 CD11b CD15 CD68 CD14 HLA-DR CD45 CD107a PGP9.5 CD49f CollagenIV ICOS aSMA Podoplanin Vimentin Gal3 RORgammaT CD38 CD45RA CD117 CD21 DAPI PanCK CD163 TFAM Ki67 FoxP3 GranzymeB CD134 TIM3 PDL1 CD31
Stanford-HNC
B cells
DCs
Lymphatics
MDSCs
Mac
Mast cells
Monocytes
Muscle
NK cells
Nerves
Neutrophils
Stromal
Teff
Th
Treg
Tumor
Vessels
CD20 PanCK CD11c CD197 Podoplanin CD49f Ki67 p16 GranzymeB CD45RA CD134 TMEM16A CD21 CD68 CD117 Gal3 HLA-DR CD14 aSMA CD57 PGP9.5 CD15 CD11b Vimentin CollagenIV PDL1 DAPI PD1 CD8 CD3e CD45 CD4 CD45RO FoxP3 GATA3 CD107a TFAM CD31 CD34
DFCI-HNC
APC/macrophage
B cell
CD4 T cell (ICOS+/FoxP3+)
CD8 T cell
Dendritic cell
Granulocyte
Macrophage
Mast cell
NK cell
Naive B cell
Naive lymphocyte (CD45RA+/CD38+)
Stroma
T cell (CD45RO+/FoxP3+/ICOS+)
T cell (GranzymeB+/LAG3+)
Tumor (CD15+)
Tumor (Ki67+)
Tumor (PanCK hi)
Tumor (PanCK low)
Unclassified
Unknown (TCF1+)
Vessel endothelium
HLA-DR CD20 CD4 CD3e CD45RO FoxP3 ICOS CD45 CD197 CD8 CD11c CD15 CD11b CD68 CD14 CD163 CD117 GranzymeK CD183 CD103 CD45RA CD38 CD69 Vimentin CD56 CD39 Siglec8 GranzymeB HLA-ABC Ki67 PanCK Perforin PDL1 PD1 DAPI TIM3 LAG3 CD25 TCF1 TCRgammadelta CD31

**Figure S7:** Heatmap showing the mean biomarker expression for each cell type identified in each study. Colors represent normalized biomarker expression (Methods), scaled from zero to one for visualization purposes.

| | Expression R2 | Cell Type F1 |
|---|---|---|
| Model 1 | 0.523 | 0.669 |
| Model 2 | 0.532 | 0.671 |
| Model 3 | 0.523 | 0.675 |
| Model 4 | 0.530 | 0.671 |
| Model 5 | 0.524 | 0.674 |
| Ensemble | 0.534 | 0.727 |

**Figure S8**: Performance of each of the 5 individual models used in Table 1, Main Panel on the UPMC-HNC dataset. Each model's performance is similar, but ensembling their predictions produces an overall higher score for both metrics.

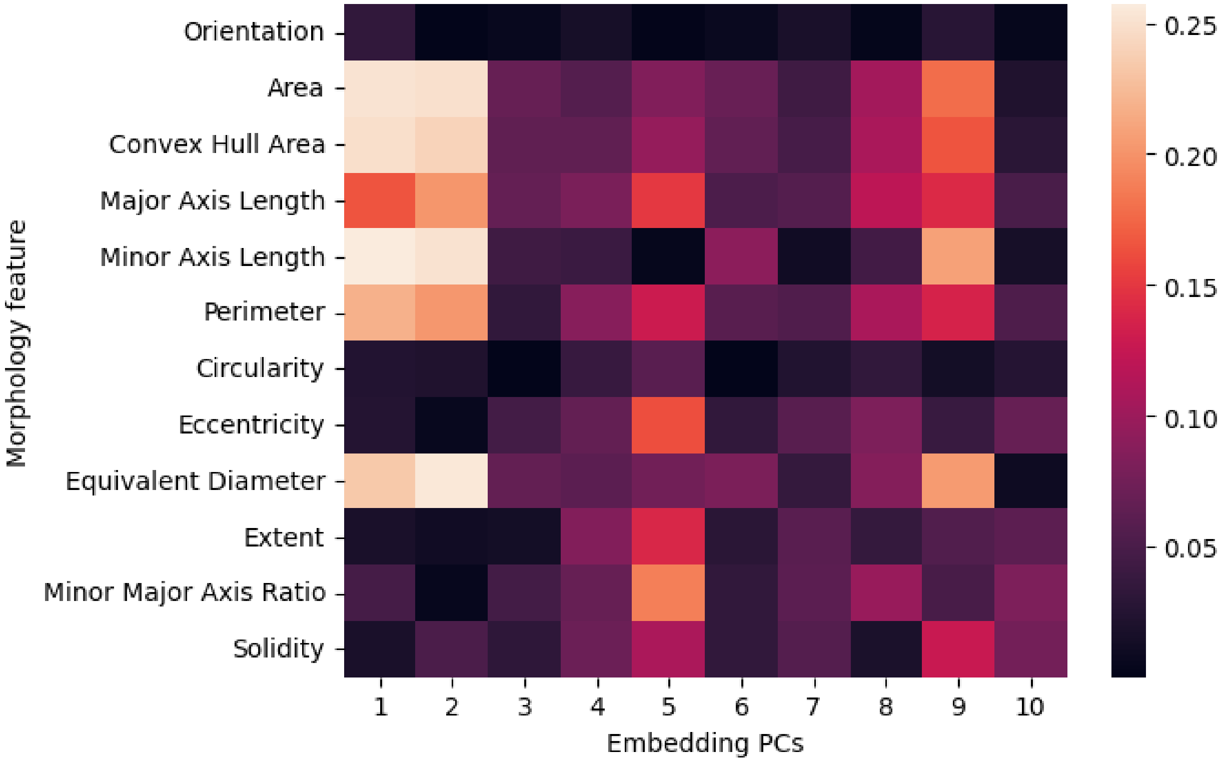

**Figure S9:** Pearson correlation matrix between twelve morphology features computed on the cell segmentation mask and the top 10 principal components (PCs) of the last embedding layer of the deep learning model using the UPMC-HNC dataset. Morphology features are defined in the Methods section.

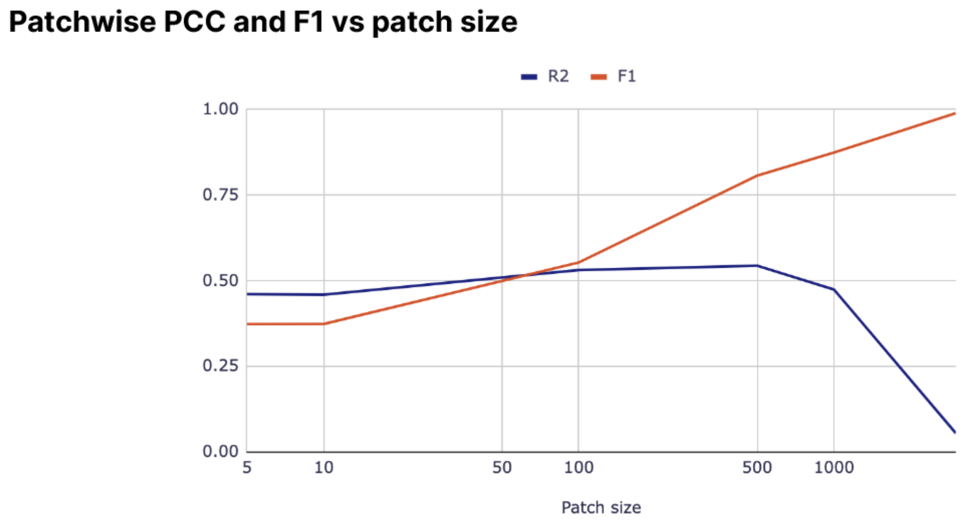


**Figure S10**: The effect of patch size (in pixels) on average patchwise PCC and F1. We use a patch size of 100 pixels when computing patchwise metrics.
